# Molecular patterns of alpha-thalassemia in the kingdom of Saudi Arabia: identification of prevalent genotypes and regions with high incidence

**DOI:** 10.1186/s12959-023-00560-w

**Published:** 2023-11-10

**Authors:** Hayaa M. Alhuthali, Eman F. Ataya, Alaa Alsalmi, Triq E Elmissbah, Khalaf F Alsharif, Hind A. Alzahrani, Ahad Amer Alsaiari, Mamdouh Allahyani, Amal F. Gharib, Husam Qanash, Heba M. Elmasry, Doha Elsayed Hassanein

**Affiliations:** 1 Department of Clinical Laboratory Sciences, College of Applied Medical Sciences, Taif University, PO Box 11099, Taif, 21944 Saudi Arabia; 2 Basic sciences, College of Applied Medical Sciences, Albaha University, Albaha, 4781 Saudi Arabia; 3 Lecturer of Public Health and Community Medicine, Faculty of Medicine, Cairo University, Giza, Egypt; 4 Department of Medical Laboratory Science, College of Applied Medical Sciences, University of Ha’il, Hail, 55476 Saudi Arabia; 5 Medical and Diagnostic Research Center, University of Ha’il, Hail, 55473 Saudi Arabia; 6 Al Borg Medical laboratories, Al Borg Diagnostics, Jeddah, Saudi Arabia; 7 Department of Clinical Pathology, South Egypt Cancer Institute, Assiut, Egypt; 8 Present Address: Misr University for Sciences and Technology, Clinical and Chemical Pathology Department, Cairo, Egypt

**Keywords:** Alpha-thalassemia genotypes, α-thalassemia in the Saudi population, Consanguineous marriages, Premarital testing program

## Abstract

**Background:**

Alpha-thalassemia (α-thalassemia) is one of the most common monogenic diseases in Saudi Arabia and is associated with significant morbidity. Premarital testing programs in Saudi Arabia reduce the burden of hemoglobinopathy disorders, and ongoing monitoring is required. We aimed to explore the molecular nature of α-globin genes and identify the most common genotypes and regions with a high risk of α-thalassemia in Saudi Arabia.

**Methods:**

This retrospective study was conducted between January 2021 and December 2022. Six hundred twenty-five samples from patients with microcytic hypochromic anemia in Saudi Arabia were analyzed using reverse dot blot hybridization (RDBH)-based multiplex-PCR, which screens for the known 21 mutations of α-globin genes.

**Results:**

Seven mutations in the α-globin gene were identified in 88.96% (556) patients. The most frequent abnormality of a-globin genes was −α^3.7^ (62.3%), followed by α2^IVS1(−5nt)^ (20.7%) and α2 polyA-1 (α2^T.Saudi^) (14.1%). Interestingly, α2 polyA-2 (α2^T.Turkish^) was identified in Saudi and presented with −MED, causing Haemoglobin H disease. The incidence of α-thalassemia in Saudi Arabia’s cities showed significant differences (P = 0.004). Jeddah City had the highest percentage of cases (25%), followed by Makkah (23%), Taif (13.3%), and Al-Ahassa (12.4%).

**Conclusion:**

The study provides current knowledge about the molecular nature of α- thalassemia, highlights the common genotypes that could contribute to disease occurrence in the Saudi population, and sheds light on Saudi regions with a high incidence. It also recommends further studies in a larger population and with differently composed molecular assays to verify these findings.

## Background

Hemoglobinopathies are the most common hereditary disease worldwide. Thalassemia and sickle cell anemia are associated with a significant impact on morbidity and mortality among affected individuals [1]. Alpha-thalassemia (α-thalassemia) is prevalent in Mediterranean countries, the Indian subcontinent, the Middle East, Southern China, and Southeast Asia [2]. In Saudi Arabia, sickle cell anemia and beta-thalassemia are 49.6 and 13.6 per 1000 population, respectively [3]. α-thalassemia is also highly prevalent, ranging from 0.4% in the Northern region to 5.9% in the Eastern region, and is co-inherited with sickle cell disease and beta-thalassemia [4, 5]. The α-thalassemia trait was recently suspected of having the highest frequency (6.3%) of hemoglobin abnormality in premarital screening in Makkah City [6].

α-thalassemia is characterized by a lack of or inadequate synthesis of one or more α-globin chains. The α-globin genes are composed of two types, HBA1 and HBA2, and are found on the 16p13.3 chromosome [7]. There is another type of α-globin gene named HBA12 discovered in the Saudi population [8]. HBA12 replaced HBA2 in 5.7% of the Saudi population. HBA12 was formed by gene conversion of HBA2 and HBA1 gene sequences [8, 9].

Typically, there are four genes (α1α2/α1α2), which correspond to four α-globin proteins. Reduction in the expression of one or more of these genes results in different α-thalassemia phenotypes, which correlates with clinical variation and disease severity [10]. α-thalassemia is classified as α^+^-thalassemia and α^0^-thalassemia depending on how many functional α-globin genes remain [11]. α^+^-thalassemia is defined by the deletion of one pair (–α/αα) of α-globin genes while α^0^-thalassemia has double deletion (–/αα). Heterozygosity of α^+^-thalassemia results in a silent carrier state, which often has no clinical symptoms and overlaps with normal red blood cell indices [12]. The homozygosity state for α^+^-thalassemia and heterozygous state for α^0^-thalassemia are clinical traits with mild microcytic and hypochromic anemia and have a normal HBA2 level [12]. The co-inheritance of these milder thalassemia forms (--/-a) causes adult hemoglobin H disease (HbH disease), which is characterized by excess β-globin chains [13]. HbH disease is associated with microcytic hypochromic chronic hemolytic anemia, mild jaundice, splenomegaly, acute hemolysis in response to infections and oxidant drugs, and the need for blood transfusions or iron chelation therapy on rare occasions [12, 14].

Four mutant copies of alpha genes cause Hb Bart’s hydrops fetalis disorder in fetuses (also called α-thalassemia major). Hydrops fetalis is characterized by γ-globin chain accumulation with a decrease in α-globin chain synthesis, and almost all affected newborns die in utero [14].

It has been reported that the number of deleted α-genes significantly correlates with mean corpuscular hemoglobin (MCH), red cell distribution width (RDW), and mean corpuscular volume (MCV). A heterogenous α^0^ allele is strongly predicted in the presence of MCV < 70.80 fL and MCH < 21.90 pg, while an α^0^ allele should be excluded in individuals with microcytosis, MCH < 23.40 pg, and normal values of HbF and HBA2 and without iron deficiency [15]. Furthermore, both MCH < 19 pg and RDW ≥ 20% strongly predict HbH disease [15].

Molecular defects of α-globin genes occur in two forms: deletion with α^0^ and α^+^ phenotypes and non-deletion abnormalities with point mutation. Deletional mutations cause more than 95% of α-thalassemia syndromes, while point mutations are causative for the rest [16]. The most prevalent deletional form of HbH disease is compound heterozygosity with a single and double globin gene deletion on opposite alleles [17]. The second type of HbH disease, non-deletional, means that at least one genetic abnormality was a non-deletional mutation [18]. This form is rare and mainly occurs due to homozygosity with non-deletional alleles. However, sometimes compound heterozygosity for a point mutation in either the globin gene 1 or 2 on one chromosome and double globin gene deletion on another chromosome can also produce non-deletional HbH [18]. Patients with non-deletional HbH disease have more serious clinical symptoms, are more anemic, are more likely to develop hepatosplenomegaly and require frequent blood transfusions [19].

Molecular screening and identification of α-thalassemia contribute to prenatal diagnosis and realistic genetic counseling in regions at thalassaemic risk. Premarital testing programs in Saudi Arabia reduce the burden of hemoglobinopathy disorders, and ongoing monitoring is required. This study aims to evaluate the molecular nature of α-globin genes in α-thalassemia patients in Saudi Arabia and identify the prevalent genotypes and distribution of the disease among Saudi cities. The study’s findings could shed light on regions with a high incidence that need more improvement in disease prevention efforts and health promotion. Also, it could help evaluate the effectiveness of genetic counseling programs in achieving a massive reduction of the disease burden.

## Methods

### Sample and population

The current study is a retrospective study that included all samples received at Al Borg Diagnostic Lab and analyzed from January 2021 to December 2022 in Saudi Arabia. Al Borg Diagnostic Lab is the largest chain of private laboratories in medical diagnostics. It has branches over all regions (Western, Eastern, Northern, Southern, and Central) of Saudi Arabia with one connected system. Six hundred twenty-five blood samples were collected from patients aged 25–75 in EDTA tubes. The samples were referred to Alborg Laboratory for molecular tests to confirm α-thalassemia diagnosis. The inclusion criteria of the samples included microcytosis, MCV < 80 fL or hypothermia, MCH < 26 pg, or both without iron deficiency anemia (IDA) [20]. Exclusion criteria included patients with iron deficiency anemia who show low RBC indices and abnormal iron profiles, including serum iron < 65/ 50 µg/dl in males and females, respectively, and ferritin < 30 ng/l that improved after a minor course of iron treatment [20]. the presence of any chronic diseases and, blood transfusion in the last three months, and pregnancy.

### Techniques/instruments

According to the commercial instructions, DNA was isolated from blood samples (QIAamp blood mini kit, Qiagen, Hilden, Germany). After DNA extraction, the patients’ DNAs were screened for mutations in the α-globin genes. Reverse dot blot hybridization (RDBH)-based multiplex-PCR was used to screen the samples for the known 21 mutations of α-globin genes (MED double gene deletion, THAI double gene deletion, SEA double gene deletion, FIL double gene deletion, 20.5 kb double gene deletion, 3.7 single gene deletion, 4.2 single gene deletion, α1 cd 14 [TGG > TAG], anti-3.7 gene triplication, α2 init cd [ATG > ACG], α2 cd 19 [-G], α2 IVS1 [-5nt], α1 cd 59 [GGC > GAC] (Hb Adana), α2 cd 59 [GGC > GAC], α2 cd 142 [TAA > AAA] (Hb Icaria), α2 cd 142 [TAA > TAT] (Hb Pakse), α2 cd 142 [TAA > CAA] (Hb Constant Spring), α2 cd 125 [CTG > CCG] (Hb Quong Sze), α2 cd 142 [TAA > TCA] (Hb Koya Dora), α2 poly A-2 [AATAAA-AATGAA], α2 poly A-1 [AATAAA-AATAAG]). Amplification products were identified by hybridization to allele-specific oligonucleotide probes as an array of parallel lines according to a commercial methodology (α-globin stripAssay, ViennaLab Diagnostics, Vienna, Austria) [21, 22].

Different α-globin genotypes were defined, and the frequencies of the alleles were calculated. The severity of the disease was categorized as silent carrier, α-thalassemia trait, or HbH disease based on the type, location, and number of the detected mutated alleles [23]. The data were analyzed using the appropriate statistical tests of SPSS, version 20.0 (SPSS Inc., Chicago, IL, USA). Quantitative data were expressed as mean ± standard deviation (SD). Qualitative data were expressed as frequencies and percentages, and a chi-square (χ^2^) test was applied. The confidence interval was set to 95%, and the significance was considered if the P-value was < 0.05.

## Results

Of the 625 patients in this study, 53.4% were female, and 46.6% were male. The average age was 29.08 ± 11.6 years. A total of seven different mutations in the α-globin gene of α-thalassemia were identified in 88.96% (n = 556) patients, and 11% (n = 69) of the analyzed chromosomes had no mutations detected (Tables 1 and 2). Table 1 presents the mutated alleles and the number of affected chromosomes based on the detected genotype. The most frequent abnormality of a-globin genes was −α^3.7^ (62.3%), followed by α2^IVS1(−5nt)^ (20.7%) and α2 polyA-1 (α2^T.Saudi^) (14.1%). −MED, −SEA, α2 polyA-2 (α2^T.Turkish^), and −α^4.2^ were the remaining molecular defects seen in 1.0% and 1.8% of patients, respectively (Table 1). The −α^3.7^/−α^3.7^ genotype was found to be the most common genotype, with 26.1%. The α^IVS1(−5nt)^ α/αα genotype was the second most common genotype with 20.6%, followed by −α^3.7^/αα (19.8%) (Table 2).

**Table 1:** The number of the affected chromosomes is based on the detected mutation

Mutation	NO. of the affected chromosome	Percent
--^SEA^	8	0.99%
--^MED^	14	1.8%
– α^3.7^	503	62.3%
- α ^4.2^	1	0.12%
α2 ^IVS1 (−5nt)^	167	20.7%
α2 polyA-1 ^(α2 T.Saudi)^	113	14.1%
α2 polyA-2 (α2^T.Turkish^)	1	0.12%
Total	807	100%

SEA: Southeast Asia, MED: Mediterranean, IVS1: intervening sequence1

**Table 2:** Genotypes and phenotypes of α-thalassemia cases

Genotype	Mutation	Phenotype	N (%)
**--** ^**SEA**^ **/ αα**	Deletional Mutation	α-thalassemia trait	8 (1.3)
**- α** ^**3.7**^ **/ α** ^**TSaudi**^ **α**	Deletional and Non-deletional Mutation	α-thalassemia trait	41 (6.6)
**- α** ^**3.7**^ **/α** ^**IVS1(−5nt)**^ **α**	Deletional and Non-deletional mutation	α-thalassemia trait	11 (1.8)
**α** ^**IVS1 (−5nt)**^ **α/ α** ^**TSaudi**^ **α**	Non-deletional mutations	α-thalassemia trait	13 (2.1)
**- α** ^**3.7**^ **/ - α** ^**4.2**^ **α**	Deletional mutation	α-thalassemia trait	1 (0.2)
**--** ^**MED/**^ **α** ^**TTurkish**^ **α**	Deletional and Non-deletional Mutation	HbH disease	1 (0.2)
**- α** ^**3.7**^ **/- α** ^**3.7**^	Deletional mutation	α-thalassemia trait	163 (26.1)
**- α** ^**3.7**^ **/ αα**	Deletional mutation	Silent carrier	124 (19.8)
**α** ^**TSaudi**^ **α/α** ^**TSaudi**^ **α**	Non-deletional mutation	α-thalassemia trait	14 (2.2)
**α** ^**TSaudi**^ **α / αα**	Non-deletional mutation	Silent carrier	31 (5)
**α** ^**IVS1 (−5nt)**^ **α / α** ^**IVS1 (−5nt)**^ **α**	Non-deletional mutation	α-thalassemia trait	7 (1.1)
**α** ^**IVS1 (−5nt)**^ **α / αα**	Non-deletional Mutation	Silent carrier	129 (20.6)
**--** ^**MED/**^ **αα**	Deletional Mutation	Alpha-thal trait	13 (2.1)
**No Mutation Detected**	69 (11.0)		
Total	625 (100)		

The distribution of genotypes among the genders was investigated and showed a significant difference (P = 0.04). For example, −α^3.7^/α^T.Saudi M^α and −α^3.7^/−α^3.7^ were found to be prominent in males (63.4%) compared to females (54.6%) (P ≤ 0.03), while −α^3.7^/α α was significantly higher in females (62.1%) than in males (37.9%) (P = 0.04) (Table 3).

**Table 3:** Distribution of the detected genotypes according to gender

Genotypes		Gender		Total	P value	P value
		Female	Male			
**No Mutation Detected**	N %	41 59.4%	28 40.6%	69 100.0%	0.31	
**--** ^**SEA**^ **/ αα**	N %	3 37.5%	5 62.5%	8 100.0%	0.48	
**- α** ^**3.7**^ **/ α** ^**TSaudi**^ **α**	N %	15 36.6%	26 63.4%	41 100.0%	0.03*	
**- α** ^**3.7**^ **/α** ^**IVS1(−5nt)**^ **α**	N %	8 72.7%	3 27.3%	11 100.0%	0.24	
**α** ^**IVS1 (−5nt)**^ **α/ α** ^**TSaudi**^ **α**	N %	5 38.5%	8 61.5%	13 100.0%	0.40	
**- α** ^**3.7**^ **/ - α** ^**4.2**^ **α**	N %	1 100.0%	0 0.0%	1 100.0%	1.0	
**--** ^**MED/**^ **α** ^**TTurkish**^ **α**	N %	1 100.0%	0 0.0%	1 100.0%	1.0	
**- α** ^**3.7**^ **/- α** ^**3.7**^	N %	74 45.4%	89 54.6%	163 100.0%	0.02*	0.04*
**- α** ^**3.7**^ **/ αα**	N %	77 62.1%	47 37.9%	124 100.0%	0.04*	
**α** ^**TSaudi**^ **α/α** ^**TSaudi**^ **α**	N %	7 50.0%	7 50.0%	14 100.0%	0.79	
**α** ^**TSaudi**^ **α / αα**	N %	20 64.5%	11 35.5%	31 100.0%	0.27	
**α** ^**IVS1 (−5nt)**^ **α / α** ^**IVS1 (−5nt)**^ **α**	N %	3 42.9%	4 57.1%	7 100.0%	0.71	
**α** ^**IVS1 (−5nt)α**^ **/ αα**	N %	74 57.4%	55 42.6%	129 100.0%	0.32	
**--** ^**MED/**^ **αα**	N %	5 38.5%	8 61.5%	13 100.0%	0.40	
**Total**	N %	334 53.4%	291 46.6%	625 100.0%	0.09	

* P < 0.05

Genotype was classified into three categories: deletional mutation, non-deletional mutation, and the combination of deletional and non-deletional mutation. These three groups of molecular defects accounted for 88.96% of the total cases (n = 556/625). As shown in Tables 4 and 55.6% of the patients had the deletional mutation, 34.9% had a non-deletional mutation, and 9.5% were found to have a combination of deletional and non-deletional mutated alleles.

In addition, Table 4 represents the distribution of α-thalassemia patients in Saudi Arabia’s cities and shows significant differences (P = 0.004). Jeddah City had the highest percentage of cases (25%), followed by Makkah (23%), Taif (13.3%), and Al-Ahssa (12.4%). The lowest frequency of the disease was found in Khobar (0.36%) and Najran and Hail (0.18%). Moreover, Makkah, Riyadh, Al-Ahssa, and Jeddah were found to have significant differences in the frequencies of the genotypes (P ≤ 0.04). Deletional mutation was the most frequent genotype in Makkah (51.6%), Riyadh (74.5%), and Al-Hassa (68.1%), while non-deletional mutation was a common genotype in Jeddah City (48.9%).

**Table 4:** Genotype frequencies and their distribution according to the Saudi cities

Cities		Genotype			Total (%)	P value	P value
		Deletional Mutation	Deletional &non-deletional mutation	Non-deletional Mutation			
**Makkah**	N %	66 51.6%	10 7.8%	52 40.6%	128 100.0%	0.04*	0.004**
**Riyadh**	N %	38 74.5%	2 3.9%	11 21.6%	51 100.0%	0.02*	
**Jeddah**	N %	61 43.8%	10 7.3%	68 48.9%	139 100.0%	0.001**	
**Medina**	N %	2 28.6%	2 28.6%	3 42.9%	7 100.0%	0.15	
**Taif**	N %	41 55.4%	7 9.5%	26 35.1%	74 100.0%	0.9	
**Jizan**	N %	14 51.9%	5 18.5%	8 29.6%	27 100.0%	0.26	
**Jubail**	N %	11 57.9%	3 15.8%	5 26.3%	19 100.0%	0.54	
**Al-Ahassa**	N %	47 68.1%	13 18.8%	9 13.0%	69 100.0%	0.001**	
**Al-Baha**	N %	6 100.0%	0 0.0%	0 0.0%	6 100.0%	0.09	
**Khamis Mushait**	N %	4 57.1%	0 0.0%	3 42.9%	7 100.0%	0.67	
**Khobar**	N %	2 100.0%	0 0.0%	0 0.0%	2 100.0%	0.45	
**Dammam**	N %	5 55.6%	0 0.0%	4 44.4%	9 100.0%	0.57	
**Tabuk**	N %	2 50.0%	1 25.0%	1 25.0%	4 100.0%	0.56	
**Abha**	N %	4 80.0%	0 0.0%	1 20.0%	5 100.0%	0.51	
**Abu Arish**	N %	4 57.1%	0 0.0%	3 42.9%	7 100.0%	0.67	
**Hail**	N %	1 100.0%	0 0.0%	0 0.0%	1 100.0%	NA*	
**Najran**	N %	1 100.0%	0 0.0%	0 0.0%	1 100.0%	NA*	
**Total**	N %	309 55.6%	53 9.5%	194 34.9%	556 100.0%		

* P < 0.05, ** P < 0.01

NA: Not applicable

## Discussion

The molecular diagnosis of α-thalassemia assists in genetic counseling and selecting patients at high risk of developing diseases. Saudi Arabia has the highest prevalence of thalassemia in the world [5]. Ongoing identification of frequent genotypes and areas with high incidence is essential. This study detected seven molecular defects of α-globin genes in the Saudi population. These are −α^3.7^, α2^IVS1(−5nt)^, α2^T.Saudi^, −MED, −SEA, −α^4.2^, and α2^T.Turkish^. −α^3.7^, α2^IVS1(−5nt)^, and α2^T.Saudi^ were the most frequent mutated alleles with 62.3%, 20.7%, and 14.1%, respectively. In addition, the current study explored the extent of the genotype’s diversity among patients with α-thalassemia. The majority of Saudi individuals had −α^3.7^/−α^3.7^ (26.1%), followed by α^IVS1(−5nt)^ α/αα (20.6%) and −α^3.7^/αα (19.8%). As previously reported [24, 25], we found that −α ^3.7^, either homozygous or heterozygous genotype, was the highest mutated allele among all the α-thalassemia Saudi patients (62.3%). This finding is also consistent with studies by Akhtar, Qaw [26] and Attallahm, Alhadad [27], who demonstrated that the homozygous and heterozygous types of −α^3.7^ were common α-thalassemia mutations in Saudi’s Eastern and Western regions, respectively. α^+^ deletion −α^3.7^ was reported as the most prevalent mutation in Iran [7, 28], Western Australia [29], Amazon [30], Northern Thailand [14], and Antalya [21].

Akhtar, Qaw [26] identified three α^0^ deletional mutations and seven-point mutations in Saudi people living in the eastern province. Among these, our study reported only α^0^ deletion −MED and α2^T Saudi^ despite the study included 99 individuals living in the eastern province of Saudi Arabia. Attallahm, Alhadad [27] have reported –SEA mutations in addition to –MED and α2^PA − 1^ in the Western province of Saudi Arabia,

α2^T.Saudi^ non-deletional allele is widespread in the Arabian Gulf. Many reports have documented α2^T.Saudi^ as the most common cause of HbH disease in Kuwait [31], Jordan [32], Bahrain [33, 34], UAE [35], and the Kingdom of Saudi Arabia [36, 37].

In this study, α2^T.Saudi^ was a common allele (14.1%) but found as either an α-thalassemia trait or a silent carrier in all detected genotypes. The non-deletional mutation α2^IVS1(−5nt)^ was detected in the Saudi population in different genotype forms (Table 2); α2^IVS1(−5nt)^ heterozygous was the common genotype. Non-gene deletional α2^IVS1(−5nt)^ has been reported to be prevalent in Arabs and has a significant impact on the genotype/phenotype correlation [38, 39].

This study also reported the presence of the −SEA and α2^T.Turkish^ alleles among Saudi patients. −SEA presented as heterozygous while α2^T.Turkish^ non-deletional allele presented as heterozygous with −MED deletional allele causing HbH disease.

Of course, the closed commercial methodology does not allow us to generalize the findings; further studies are required in a larger population and with differently composed molecular assays to verify these findings. HBA12 is an HBA2 gene convert discovered in the Saudi population [8]. The α-globin gene was reported to be co-inherited with α-globin gene defects [9]. Mutations on the ATRX gene (Cd39(C→T), IVS I-5(G→C), c.848T > C, and c.623delA) were found to contribute to α-thalassemia-like phenotype and have been reported in the Saudi population [9]. Molecular screening of ATRX and HBA12 genes, along with the analysis of HBA1 and HBA2, is recommended for proper estimation of the disease burden in the Saudi population.

Gender can affect disease prognosis and is increasingly crucial in medical fields. Several studies have demonstrated that thalassemia is linked with various gender differences [40–43]. Marsella, Pepe [44] demonstrated that females have better and longer survival rates than males with thalassemia. The current study investigated the distribution of the α-thalassemia genotypes among genders and found significant differences (P = 0.04). The frequency of −α^3.7^ homozygous and the combined heterozygous −α^3.7^ and α2^T.Saudi^ hemizygous was more significant in males (P ≤ 0.02) than females, while −α^3.7^ heterozygous was significantly higher in females (P = 0.04). Similar to our findings, a study performed in Brazil [30] found that males (5.89%) showed a slightly greater frequency of −α^3.7^ than females (4.0%). However, Husna, Sanka [45] demonstrated that the frequency of thalassemia carriers was more significant in females than males. In addition, another study conducted to examine the effect of gender among non-transfusion-dependent thalassemia patients showed that females were more anemic than males. However, there were no significant differences related to the disease complication [46], which may highlight the need to follow up with pregnant females with thalassemia carriers or traits and provide effective genetic counseling.

A higher prevalence of hemoglobinopathies was found in the western province of Saudi Arabia [47]. Consistently, in his study, the analysis of α-thalassemia distribution among Saudi cities found that western province cities, including Jeddah, Makkah, and Taif, had the highest percentage (61.3%) of α-thalassemia patients. In comparison to Riyadh, which has a high population (approximately 5–6 million) similar to what Jeddah has, a low frequency of α-thalassemia was found (9%) while Jeddah had a high incidence (25%). Furthermore, the higher prevalence of α-thalassemia mutations in Makkah (23%) and Taif (13.3%), which are considered small cities with less population (≤ 2 million), indicates that people in these areas are at high risk for developing the disease. Moreover, consistent with previous findings [25], among the eastern province cities, Al-Ahssa was found to have a higher frequency of α-thalassemia (12.4%).

Point mutations have been reported to be more prevalent than large deletions among Arabs [24]. In contrast, in the current study, the deletional mutations were most common and seen in about half of the population (55.6%), followed by the non-deletion (34.9%) and then the combined heterozygous of deletional and non-deletional alleles (9.5%). The deletional mutations were significantly higher in Makkah, Riyadh, and Al-Ahssa (P ≤ 0.04), whereas the non-deletional alleles were the most frequent in Jeddah (P = 0.001).

Consanguinity is high in Saudi Arabia and is estimated to be 56%, which is higher in rural areas [48]. The Healthy Marriage Programme is a mandatory screening established in 2004. The program screens couples planning to get married for common hereditary disorders, including thalassemia. The program successfully screens the most eligible individuals, reducing the number of high-risk marriages and lowering the frequency of HbH diseases in generations.

This study has many limitations that need to be addressed. First, power and sample size were not calculated. Second, as it is a retrospective study, the data are limited to the laboratory’s resources. For instance, the molecular nature of α-thalassemia was restricted to the diagnostic assay proved in the Alborg Diagnostic Lab, which assesses only the known 21 mutations of α-globin genes. Also, because of the limited laboratory record, hematological indices could not be analyzed. Third, the data obtained in this study were from a private diagnostic laboratory, and we expect most of the included participants were a couple planning to get married; thus, most of the cases in our study were α-thalassemia traits or carriers. Hence, the study may underestimate the severity of α-thalassemia in the Saudi population. In addition, because Alborg Diagnostic Laboratory is located in the major cities of Saudi Arabia, this could lead to an underestimation of the prevalence of α-thalassemia in rural areas. Finally, the study missed the silence α-thalassemia group with normal RBC, MCV, and MCH, which may also underestimate the incidence of the disease.

## Conclusion

The current study confirmed the diversity of α-thalassemia genotypes among the Saudi population and highlighted the most common alleles in a selected population. It demonstrated that the −SEA and α^T.Turkish^ alleles are also encountered in Saudi populations. The findings of this study might be used to develop recommendations for diagnosing and managing thalassemia and to enhance public health services in Saudi Arabia. It suggests that educational programs and health campaigns should be improved mainly in regions with a higher risk to expand the population’s knowledge about the disease and consequences of consanguineous marriages. At-risk couples must achieve a comprehensive awareness of the seriousness of the disease before they decide to get married through a more efficient genetic counseling program to achieve the most significant reduction rate for this disorder. Further studies in a larger population and at a large scale to include all genes that could contribute to the disease occurrence are recommended to verify these findings and include other prevalent mutations.

## Data Availability

All data that support the study’s findings is contained within the manuscript.

## References

[1] Weatherall DJ (2011). The challenge of haemoglobinopathies in resource-poor countries. Br J Haematol.

[2] Huang H, Chen M, Chen L, Zhang M, Wang Y, Lin N (2021). Prenatal diagnosis of Thalassemia in 695 pedigrees from southeastern China: a 10-year follow‐up study. J Clin Lab Anal.

[3] Alsaeed ES, Farhat GN, Assiri AM, Memish Z, Ahmed EM, Saeedi MY (2018). Distribution of hemoglobinopathy disorders in Saudi Arabia based on data from the premarital screening and genetic counseling program, 2011–2015. J Epidemiol Glob Health.

[4] Memish ZA, Owaidah TM, Saeedi MY (2011). Marked regional variations in the prevalence of sickle cell Disease and beta-thalassemia in Saudi Arabia: findings from the premarital screening and genetic counseling program. J Epidemiol Glob Health.

[5] Olwi DI, Merdad LA, Ramadan EK (2018). Thalassemia: a prevalent Disease yet unknown term among college students in Saudi Arabia. J Community Genet.

[6] Moustafa AZ, EI RAA, RA Q, ZS Q (2022). The prevalence of hemoglobin abnormality in the premarital screening Saudi population in Makkah city in a cross-sectional study. SMHJ [Internet].

[7] Dehbozorgian J, Moghadam M, Daryanoush S, Haghpanah S, Imani fard J, Aramesh A (2015). Distribution of alpha-thalassemia mutations in Iranian population. Hematology.

[8] Borgio JF, AbdulAzeez S, Al-Nafie AN, Naserullah ZA, Al-Jarrash S, Al-Madan MS (2014). A novel HBA2 gene conversion in cis or trans: alpha12 allele in a Saudi population. Blood Cells Mol Dis.

[9] Borgio JF (2015). Molecular nature of alpha-globin genes in the Saudi population. Saudi Med J.

[10] Qiu Q-W, Wu D-D, Yu L-H, Yan T-Z, Zhang W, Li Z-T (2013). Evidence of recent natural selection on the southeast Asian deletion (--SEA) causing α-thalassemia in South China. BMC Evol Biol.

[11] Munkongdee T, Vattanaviboon P, Thummarati P, Sewamart P, Winichagoon P, Fucharoen S (2010). Rapid diagnosis of α-Thalassemia by melting curve analysis. J Mol Diagn.

[12] Vichinsky EP (2013). Clinical manifestations of alpha-thalassemia. Cold Spring Harb Perspect Med.

[13] Ataulfo Gonzalez F, Blazquez C, Ropero P, Briceno O, Alaez C, Polo M (2005). [Association of hemoglobinopathy and alpha thalassemia. Study of 45 patients]. Med Clin (Barc).

[14] Lithanatudom P, Khampan P, Smith DR, Svasti S, Fucharoen S, Kangwanpong D (2016). The prevalence of alpha-thalassemia amongst Tai and Mon-Khmer ethnic groups residing in northern Thailand: a population-based study. Hematology.

[15] Velasco-Rodriguez D, Blas C, Alonso-Dominguez JM, Vega G, Soto C, Garcia-Raso A et al. Cut-off values of hematologic parameters to predict the number of alpha genes deleted in subjects with Deletional Alpha Thalassemia. Int J Mol Sci. 2017;18(12). 10.3390/ijms18122707 PMC5751308 29236053

[16] Pornprasert S, Salaeh NA, Tookjai M, Punyamung M, Pongpunyayuen P, Treesuwan K (2018). Hematological Analysis in Thai samples with Deletional and Nondeletional HbH Diseases. Lab Med.

[17] Chui DH, Fucharoen S, Chan V. Hemoglobin H Disease: not necessarily a benign disorder. Blood. 2003. 10.1182/blood-2002-07-1975 12393486

[18] Origa R, Sollaino M, Giagu N, Barella S, Campus S, Mandas C (2007). Clinical and molecular analysis of haemoglobin H Disease in Sardinia: haematological, obstetric and cardiac aspects in patients with different genotypes. Br J Haematol.

[19] Kanavakis E, Papassotiriou I, Karagiorga M, Vrettou C, Metaxotou-Mavrommati A, Stamoulakatou A (2000). Phenotypic and molecular diversity of haemoglobin H Disease: a Greek experience. Br J Haematol.

[20] Bajwa H, Basit H. Thalassemia. StatPearls. Treasure Island (FL) ineligible companies. Disclosure: Hajira Basit declares no relevant financial relationships with ineligible companies.2023.

[21] Keser I, Mercan T, Bilgen T, KÜPESİZ O, Arikan Y, Canatan D. Investigation of alpha globin gene mutations by complementary methods in Antalya. Eastern J Med. 2021;26(1).

[22] Puehringer H, Najmabadi H, Law HY, Krugluger W, Viprakasit V, Pissard S (2007). Validation of a reverse-hybridization StripAssay for the simultaneous analysis of common alpha-thalassemia point mutations and deletions. Clin Chem Lab Med.

[23] Sabath DE (2017). Molecular diagnosis of Thalassemias and hemoglobinopathies: an ACLPS critical review. Am J Clin Pathol.

[24] Hellani A, Fadel E, El-Sadadi S, El-Sweilam H, El-Dawood A, Abu-Amero KK (2009). Molecular spectrum of α-thalassemia mutations in microcytic hypochromic anemia patients from Saudi Arabia. Genetic Test Mol Biomarkers.

[25] Borgio JF, Abdulazeez S, Almandil NB, Naserullah ZA, Al–Jarrash S, Al–Suliman AM (2018). The–α3. 7 deletion in α–globin genes increases the concentration of fetal hemoglobin and hemoglobin A2 in a Saudi Arabian population. Mol Med Rep.

[26] Akhtar MS, Qaw F, Borgio JF, Albuali W, Suliman A, Nasserullah Z (2013). Spectrum of alpha-thalassemia mutations in transfusion-dependent beta-thalassemia patients from the Eastern Province of Saudi Arabia. Hemoglobin.

[27] Attallahm S, Alhadad M, Alqarni W, Filfilan D, Zaharani A, Alharby A, MOLECULAR SPECTRUM OF ALPHA THALASSEMIA MUTATIONS IN THE WESTERN PROVINCE OF SAUDI ARABIA AND RECOMMENDATION FOR PREMARITAL SCREENING. Hemasphere. 2023; 8;(7).

[28] Moradi K, Aznab M, Azimi A, Biglari M, Shafieenia S, Alibakhshi R (2022). α-thalassemia mutations in Ilam Province, West Iran. Hemoglobin.

[29] Prior J, Bittles A, Erber W (2004). A community profile of alpha thalassaemia in Western Australia. Public Health Genomics.

[30] Anselmo FC, Ferreira NS, Mota AJd, Gonçalves MS, Albuquerque SRL, Fraiji NA et al. Deletional alpha-thalassemia alleles in amazon blood donors. Advances in Hematology. 2020;2020. 10.1155/2020/4170259 PMC7178540 32351571

[31] Adekile A, Sukumaran J, Thomas D, D’Souza T, Haider M (2020). Alpha Thalassemia genotypes in Kuwait. BMC Med Genet.

[32] Ghoush MWA (2008). Subtypes of alpha thalassemia diagnosed at a Medical Center in Jordan. TSK Koruyucu Hekimlik Bülteni.

[33] Jassim N, Al-Arrayed S, Gerard N, Al-Mukharraq H, Al-Ajami A, Ducrocoq R (2001). Molecular basis of α-thalassemia in Bahrain. Bahrain Med Bull.

[34] Al Moamen NJ, Thabet A, Mahdi F, Newton H, Salman E (2018). Various α-thalassemia genotype combinations of the saudi-type polyadenylation signal mutation (αT-Saudiα) in the population of Bahrain: an update of genotype-phenotype analyses. Hemoglobin.

[35] El-Kalla S, Baysal E (1998). α-thalassemia in the United Arab Emirates. Acta Haematol.

[36] Al-Awamy BH (2000). Thalassemia syndromes in Saudi Arabia. Saudi Med J.

[37] ABDULLA ALHARBY M, ATTALLAH S, WALAA AQ, MARWA AH, FILFILAN D (2022). Characterization of the Molecular Spectrum of a-Thalassemia mutations in the Western Province of Saudi Arabia and recommendation for Premarital Screening. Med J Cairo Univ.

[38] Baysal E (2011). α-Thalassemia syndromes in the United Arab Emirates. Hemoglobin.

[39] Farra C, Badra R, Fares F, Muwakkit S, Dbaibo G, Dabbous I (2015). Alpha thalassemia allelic frequency in Lebanon. Pediatr Blood Cancer.

[40] Kyriakou A, Savva SC, Savvides I, Pangalou E, Ioannou YS, Christou S (2008). Gender differences in the prevalence and severity of bone Disease in Thalassaemia. Pediatr Endocrinol Rev.

[41] Toumba M, Skordis N (2010). Osteoporosis syndrome in Thalassaemia major: an overview. J Osteoporos.

[42] Ishikawa I, Maeda K, Nakai S, Kawaguchi Y (2000). Gender difference in the mean age at the induction of hemodialysis in patients with autosomal dominant polycystic Kidney Disease. Am J Kidney Dis.

[43] Woldu DO, Haile ZT (2015). Gender roles and perceptions of Malaria risk in agricultural communities of Mwea Division in Central Kenya. Women Health.

[44] Marsella M, Pepe A, Borgna-Pignatti C (2010). Better survival and less cardiac morbidity in female patients with Thalassemia major: a review of the literature. Ann N Y Acad Sci.

[45] Husna N, Sanka I, Al Arif A, Putri C, Leonard E, Handayani N (2017). Prevalence and distribution of Thalassemia trait screening. J Med Sci.

[46] Marsella M, Ammirabile M, Di Matola T, Pepe A, Costantini S, Filosa A (2018). Is there a difference in phenotype between males and females with non-transfusion-dependent thalassemia? A cross-sectional evaluation. Hematology.

[47] Al Jaouni SK (2010). Prevalence of Thalassemia disorders and hemoglobinopathies in Jeddah, Western Saudi Arabia. J Appl Hematol.

[48] Al-Shroby WA, Sulimani SM, Alhurishi SA, Bin Dayel ME, Alsanie NA, Alhraiwil NJ (2021). Awareness of Premarital Screening and genetic counseling among saudis and its Association with Sociodemographic Factors: a National Study. J Multidiscip Healthc.

